# Resistance of endotracheal tubes measured after extubation in ICU patients

**DOI:** 10.1186/2197-425X-3-S1-A385

**Published:** 2015-10-01

**Authors:** L Baboi, H Penet, A Stoian, H Yonis, F Gobert, F Bayle, V Leray, R Tapponnier, J-C Richard, C Guérin

**Affiliations:** Hôpital de la Croix Rousse, Lyon, France; Ecole des Haute Etudes Ingenieur, Lille, France; INSERM UMR 955, Créteil, France

## Introduction

Increase in resistance of endotracheal tube (RETT) during mechanical ventilation in ICU should reflect reduction in internal diameter due to accumulation of secretions.

## Objectives

The aim of this study was to measure RETT after extubation in ICU patients. Our hypothesis was that RETT increased with the length of use of invasive mechanical ventilation.

## Methods

The study was performed over patients intubated for at least 1 day in our ICU. Once the patient was extubated, tube was immediately stored in a plastic bag at room temperature and kept in a safe place until bench assessment. This was performed maximal 24 hours after extubation as follows. The endotracheal tube was attached to a filter (Hygrobac) and both were set to ASL 5000 active servo lung (IngMar medical). The lung model was set in passive condition in order to deliver two consecutive breaths at constant flow from 2 to -2 L/s. The filter was tested first then the filter and the endotracheal tube were run. The relationship of pressure (P) to flow was fitted to the following equation P=K_1_ flow + K_2_ flow _2_ the where K_1_ and K_2_ are constants. P pertaining to endotracheal tube was obtained by subtracting P from filter to P from filter and endotracheal tube. Dividing P by flow led to RETT=K_1_+K_2_ flow. RETT at 1 L/s (cm H2O) was equal to K_1_ + K_2_. The relationships of K1, K2 or RETT to lenght of intubation was analysed by linear mixed model where tube brand and size were factors with random effects.

## Results

We included 52 patients (34 male) of median (1st-3rd quartiles) age 68 (61-78) years. The median duration of intubation was 5.5 (2-9) days (min 1 - max 19 days). Endotracheal tubes were from Mallinckrodt (n = 45), TaperGuard (n = 4) or Rush (n = 3) brands and internal diameter 7.0 (n = 10), 7.5 (n = 39), 8.0 (n = 3) mm. The relationships of K1, K2 or RETT to length of intubation were not significant taking into account both brand and size of endotracheal tubes (figure).

## Conclusions

Increase in resistance of endotracheal tube used in the ICU is not related to the length of tracheal intubation.

**Figure 1:**
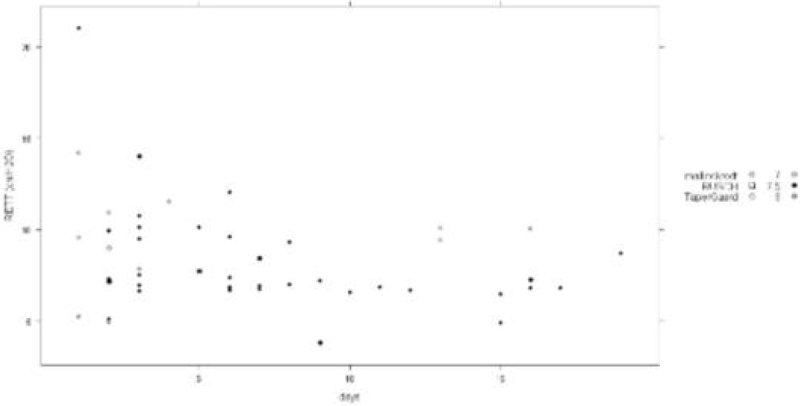
Figure 1

